# Synthesis of spiroannulated and 3-arylated 1,2,4-trioxanes from mesitylol and methyl 4-hydroxytiglate by photooxygenation and peroxyacetalization

**DOI:** 10.3762/bjoc.6.61

**Published:** 2010-06-07

**Authors:** Axel G Griesbeck, Lars-Oliver Höinck, Jörg M Neudörfl

**Affiliations:** 1 University of Cologne, Department of Chemistry, Organic Chemistry, Greinstr. 4, D-50939 Köln, Germany; Fax: +49(0) 221 470 5057

**Keywords:** ene reaction, peracetalization, peroxides, singlet oxygen, trioxanes

## Abstract

Cycloalkanones were utilized in the Lewis acid catalyzed peroxyacetalization of ß-hydroperoxy homoallylic alcohols (prepared by the ene reaction of the allylic alcohols mesitylol and methyl 4-hydroxytiglate, respectively, with singlet oxygen) to give spiroannulated 1,2,4-trioxanes **5a**–**5e** and **9a**–**9e**, respectively. A second series of 3-arylated trioxanes **10a**–**10h**, that are available from the hydroperoxy alcohol **4** and benzaldehyde derivatives, was investigated by X-ray crystallography.

## Introduction

**Figure 1:**
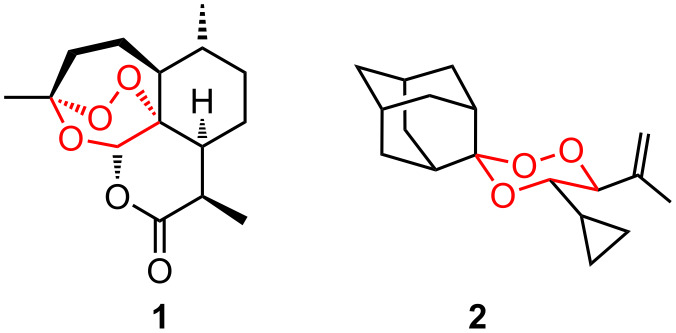
Antimalaria active natural artemisinin **1** and the spirobicyclic 1,2,4-trioxane derivative **2** show the same in vitro activity.

The antimalaria-active molecule *artemisinin* (**1**) is a naturally occurring sesquiterpene peroxide with remarkable pharmacological properties. Hydrophilic as well as lipophilic derivatives have been prepared from artemisinin and show improved antimalarial properties and better bioavailabilities [1–5]. In recent years, additional medicinal properties of artemisinin and the water soluble artesunates have been discovered such as activities against several cancer cell lines, schistosomiasis and antiviral properties [6–7]. The introduction of substituents into the central peroxide ring system as well as further ring annulation are straightforward approaches for the preparation of other active derivatives which might show promise in overcoming the forthcoming problem of artemisinin resistance [8]. From a synthetic point of view, the preparation of the pharmacophore, the central 1,2,4-trioxane ring system, is possible by a number of strategies [9–10]. We, for example, have previously reported the use of the singlet oxygen ene reaction of allylic alcohols as a route to ß-hydroperoxy alcohols that can be transformed into 1,2,4-trioxanes by reaction with carbonyl compounds in the presence of Lewis acids [11]. This approach leads to simple cyclic peroxides (e.g. **2**) which in some cases show similar antimalarial effects as the natural compound (Figure 1) [12]. An apparently useful structural feature is a large 3,3-spirofused hydrophobic group. The adamantane skeleton is a unique motif in other cyclic peroxides with antimalarial activities [13–14] which additionally exhibit other remarkable pharmaceutical properties [15–17]. In this publication we report the use of the alcohols **3** and **6** to explore further the synthetic approach to spirocyclic fused 1,2,4-trioxanes with a series of other spirofused ring structures.

## Results and Discussion

### 3,3-Spiroannulated 1,2,4-trioxanes

**Scheme 1:**
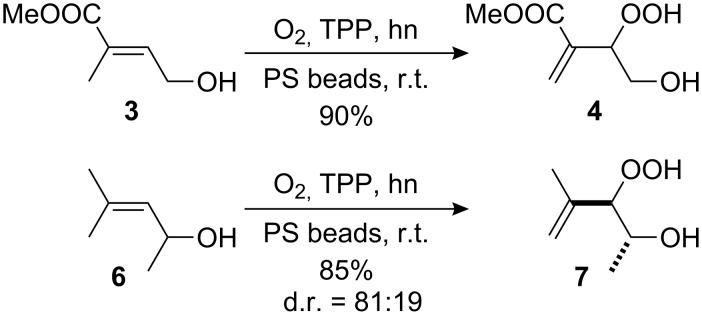
Singlet oxygen ene reaction of methyl 4-hydroxytiglate (**3**) and mesitylol (**6**) under solid-phase conditions.

**Scheme 2:**
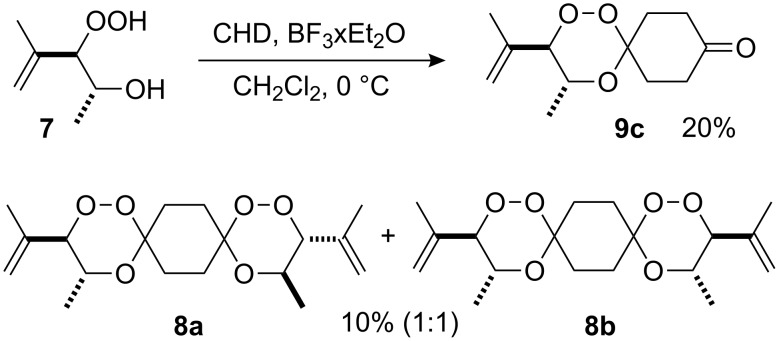
1,2,4-trioxane **9c** and *bis*-trioxane **8a**,**b** formation from the bifunctional cyclohexa-1,4-dione.

The photooxygenation reactions via sensitization of triplet oxygen with *meso*-tetraphenylporphyrin (TPP) were performed in polystyrene beads under solvent-free conditions (Scheme 1) [18–19]. Numerous applications of the hydroperoxides **4** and **7**, that result from the singlet oxygen ene reactions, have already been reported [20–21]. In context with our work on *bis*-peroxide synthesis from bifunctional ketones [22], we have also studied the peroxyacetalization of the allylic hydroperoxide **7** with the bifunctional cyclohexane-1,4-dione (CHD, Scheme 2). In this case, one equivalent of the diketone gave the monoadduct **9c** in 20% yield.

**Figure 2:**
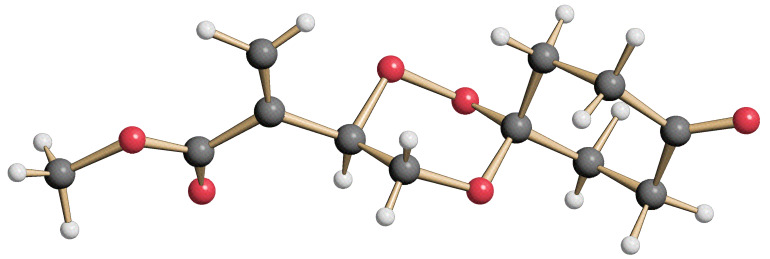
Structure of the spirobicyclic trioxane **5c** in the crystal.

The products from the reaction of monofunctional ketones with ß-hydroperoxy alcohols **4** and **7** are collected in Table 1. All trioxanes **5a**–**e** derived from **4** were crystalline and could be analyzed by X-ray structure analysis (Figure 2). The bond lengths of the crucial O-O bond were similar in all cases with the exception of the adamantane derivative **5d** which has a remarkably shorter O-O bond distance.

**Table 1:** 3,3-Spiroannulated 1,2,4-trioxanes by photooxygenation and peroxyacetalization.^a^

tiglate-derived trioxanes		Yield [%]^b^ O-O [Å]^c^	mesitylol-derived trioxanes		Yield [%]^b^ O-O [Å]^c^

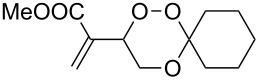	**5a**	86 1.465^d^	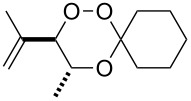	**9a**	73^e^
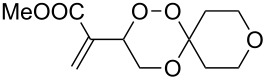	**5b**	12 1.480	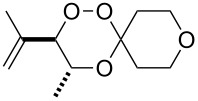	**9b**	14
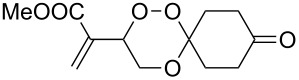	**5c**	20 1.466	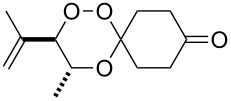	**9c**	20
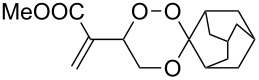	**5d**	30 1.427^d^	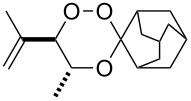	**9d**	40 1.482^f^
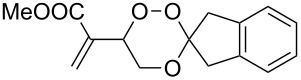	**5e**	5 1.480	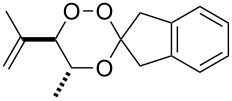	**9e**	19 1.464

^a^Standard reaction conditions: substrate (2 mmol, 4 × 10^−2^ M), CCl_4_ (50 mL), *meso*-tetraphenylporphyrin (0.01 mmol, 2 × 10^−4^ M), r.t., 10 h; then addition of a solution of the carbonyl compound (2.5 mmol) in CH_2_Cl_2_ (10 mL), 0 °C, 3 h. ^b^Yields of per-oxyacetalization. ^c^From X-ray analysis, CCDC deposited [23]. ^d^[19]. ^e^[20]. ^f^[12].

### 4-Arylated 1,2,4-trioxanes

**Scheme 3:**
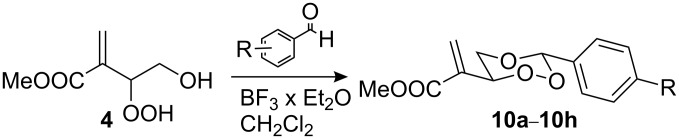
BF_3_-catalyzed acetalization of hydroperoxide **4** with benzaldehyde derivatives.

**Figure 3:**
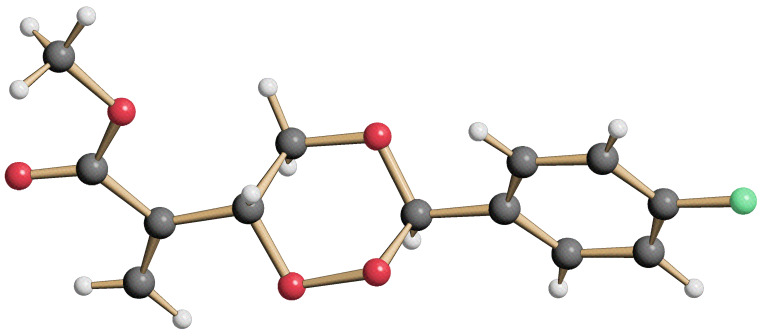
Structure of the 3-arylated trioxane **10b** in the crystal.

**Figure 4:**
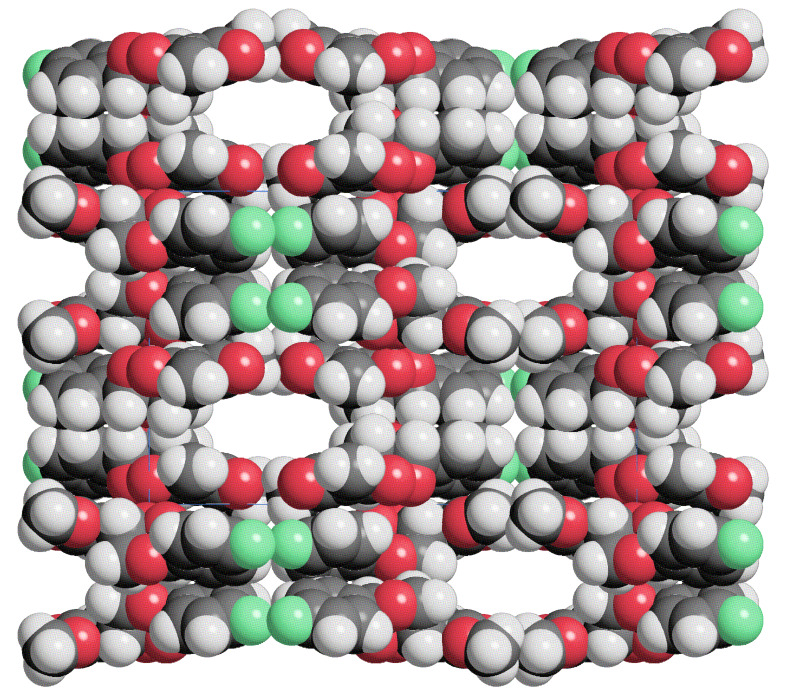
Structure of the p-bromophenyl derivative **10d** in the crystal lattice (disordered water molecules in the cannels are not shown) viewed along the a axis.

The 1,2,4-trioxanes **10** were formed in moderate to good yields, with the Hock-type cleavage product from the ß-hydroperdiol as the only side-product, from **4** and substituted benzaldehydes under BF_3_-catalysis in CH_2_Cl_2_ solution (Scheme 3). In all cases the *trans* products were formed in high (>98:2) diastereoselectivities. All compounds could be crystallized from acetone or from the neat liquid. In the crystal the central 1,2,4-trioxane ring is almost undistorted in a cyclohexane chair conformation with the acrylate and the aryl substituents in equatorial positions (Figure 3). In the crystal lattice the compounds, especially the 4-halophenyl-substituted trioxanes, tend to form π-stacked stabilized chain structures with channels that are filled with water molecules (Figure 4). In the elementary cell of the 4-chloro derivative **10c**, an average of 320 Å^3^ of channel space corresponds to one water molecules per trioxane molecule. By contrast, the 4-trifluormethyl derivative **10f** crystallized in a compact chain-like package of anti-parallel arranged pairs of trioxanes.

**Figure 5:**
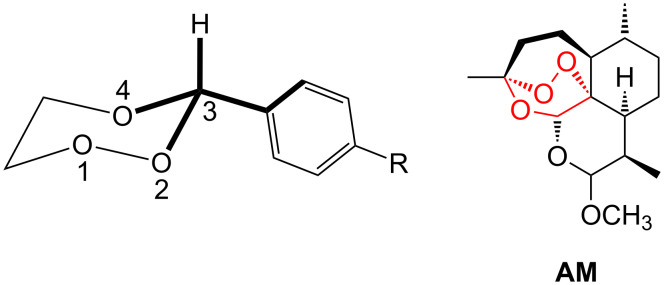
Numbering of 3-aryl-1,2,4-trioxanes **10** and relevant bonds; structure of artemether (**AM**).

The orientation of the aryl groups relative to the 1,2,4-trioxane equator depends largely on the nature of the para-substituent: in the phenyl-substituted trioxane **10a** and in the para-halogenated analogs **10b**–**10d**, the aryl group is nearly coplanar with the C(3)-H bond, whereas in the 4-nitro-, 4-trifluoromethyl-, and 4-cyano compounds **10e**–**10f** coplanarity of the aryl substituent with the O(4)-C(3) bond of the trioxane chair was observed (Table 2 and for numbering Figure 5).

**Table 2:** Structural features of the 1,2,4-trioxanes **10a**–**h**.^a^

**10**	R =	Θ_4-3-C(ar/q)-C(ar)_ (°)	Θ_2-3-C(ar/q)-C(ar)_ (°)	Θ_H(C3)-3-C(ar/q)-C(ar)_ (°)

**10a**	H	127	115	3
**10b**	F	142	100	19
**10c**	Cl	141	101	18
**10d**	Br	140	100	18
**10e**	NO_2_	179	59	59
**10f**	CF_3_	154	90	26
**10g**	CN	153	89	29
**10h**	OMe	139	103	17

^a^See [24] for CCDC submission.

In the artemisinin-derived arthemether (**AM**), the central trioxane ring has a twist-boat conformation resulting from the additional propylene bridge connecting C-3 and C-6. In Table 3 the yields of the peroxyacetalization reactions, the characteristic ^13^C NMR shifts of the peracetal carbon C-3 and two significant bond lengths are listed. It is clear that the electronic nature of the substituent on the aryl group does not significantly change the bond length of the central peroxide bond (mean value: 1.479 Å). The mean value of the characteristic ^13^C NMR shift of the peroxyacetal carbon C-3 is 103.4 ppm. The bond length of the central oxygen-oxygen bond in arthemether as determined by an independent structure analysis is 1.472(1) Å.

**Table 3:** Yields, structural and ^13^C-NMR properties of 1,2,4-trioxanes **10a**–**h**, and arthemether (**AM**).

**10**	R =	yield (%)^a^	δ(C-3) (ppm)^b^	O1-O2 (Å)	O2-C3 (Å)

**10a**^c^	H	61	104.2	1.485(7)	1.451(8)
**10b**	F	40	103.5	1.472(3)	1.432(3)
**10c**	Cl	35	103.4	1.474(3)	1.425(4)
**10d**	Br	29	103.4	1.469(9)	1.415(11)
**10e**^c^	NO_2_	31	102.6	1.471(9)	1.398(11)
**10f**	CF_3_	44	103.1	1.474(2)	1.432(2)
**10g**	CN	38	102.7	1.4823(14)	1.436(2)
**10h**	OMe	23	104.0	1.4806(19)	1.438(2)
**AM**	–	–	102.9	1.472(1)	1.416(3)

^a^Isolated yield after purification by column chromatography. ^b^In ppm, 75 MHz in CDCl_3_. ^c^Medium quality crystals, data not deposited.

More pronounced bond lengths effects were observed for the O2-C3 ring bonds that range from 1.39 to 1.45 Å. Analysis of the *Cambridge crystallographic data file* revealed that the mean oxygen-oxygen (O1-O2) bond distance for 1,2,4-trioxanes (108 compounds) is 1.472 Å with a narrow distribution ranging from the extremes 1.460 (3 compounds) to 1.482 (4 compounds). All compounds **10a**–**h** investigated by us fall into this range, **10a**,**g**,**h** showing the longest O1-O2 bond distances. With regards to antimalarial activity, all 4-arylated 1,2,4-trioxanes exhibited low in vitro activities (EC_50_/*plasmodium falciparum* > 50 μM) with the nitro-substituted compound **10e** as the most active derivative (EC_50_ = 48 μM) [25]. Thus, the peroxide bond lengths do not correlate with biological activity, cf. the highly active **AM** and the fluoro compound **10b**.

## Conclusion

In summary, we have reported the synthesis of a series of six-membered ring 3,3-spiroannulated 1,2,4-trioxanes from methyl 4-hydroxytiglate and from mesitylol, respectively, by the singlet oxygen ene reaction and subsequent peroxyacetalization. A series of 4-arylated 1,2,4-trioxanes from methyl 4-hydroxytiglate was obtained by the same protocol. These compounds were fully characterized by spectroscopic methods and by X-ray structure determination.

## Experimental

Synthesis of the 4-fluorophenyl derivative **10b**: A solution of 290 mg (2.0 mmol) of the hydroperoxide **4** (prepared from methyl 4-hydroxytiglate (**3**) by the method described in [10]) and 220 mg (2.0 mmol) of 4-fluorobenzaldehyde in 40 ml of dichloromethane was treated at 0 °C with 0.2 ml of boron trifluoride in diethyl ether. After stirring overnight at room temperature, the solution was diluted to 100 ml with dichloromethane, washed successively with 20 ml of saturated aqueous sodium bicarbonate solution, brine and water. The organic phase was separated and dried. After evaporation and column chromatography (silica, EtOAc), 200 mg (40%) of **10b** was obrained as a colorless viscous oil that crystallized as thin plates on standing: C_13_H_15_FO_6_ (corresponds to C_13_H_13_FO_5_ × H_2_O: colorless thin needles from aqueous acetone), *M* = 286.25, *a* = 6.1264(3), *b* = 16.8514(9), *c* = 26.2519(14), α, β, γ = 90°, orthorhombic, space group Pnaa, Mo-K_α_, 15276 reflections measured, 2948 reflections with *I* > 2σ(*I*), *R*_1_ (all data) = 0.0573, *wR*_2_ = 0.1811.
